# Supplementary material for: Investigating the influence of physiologically relevant hydrostatic pressure on CHO cell batch culture
Source: Sci Rep. 2021 Jan 8;11:162. doi: 10.1038/s41598-020-80576-8 (PMC7794228; doi:10.1038/s41598-020-80576-8)
Supplement: Supplementary file 1 — Supplementary Information 1. [file 41598_2020_80576_MOESM1_ESM.zip › S1/FLEX2/13Jan 30mmHg SS1 is C/Jan 12 P.pdf]

Data File C:\Users\P...hemStation\1\Data\TK_CHO_IgG 2019-01-13 17-23-06\005-5-ML Jan12 P.D
Sample Name: ML Jan12 P

===

Acq. Operator : SYSTEM Seq. Line : 5
Sample Operator : SYSTEM
Acq. Instrument : HPLC-0039 Location : 5
Injection Date : 1/13/2019 5:41:52 PM Inj : 1
Inj Volume : 100.000 µl
Sequence File : C:\Users\Public\Documents\ChemStation\1\Data\TK_CHO_IgG 2019-01-13 17-23-06\TK_CHO_IgG.S
Method : C:\Users\Public\Documents\ChemStation\1\Data\TK_CHO_IgG 2019-01-13 17-23-06\CHO_IgG_LC.M (Sequence Method)
Last changed : 12/4/2018 1:38:41 PM by SYSTEM

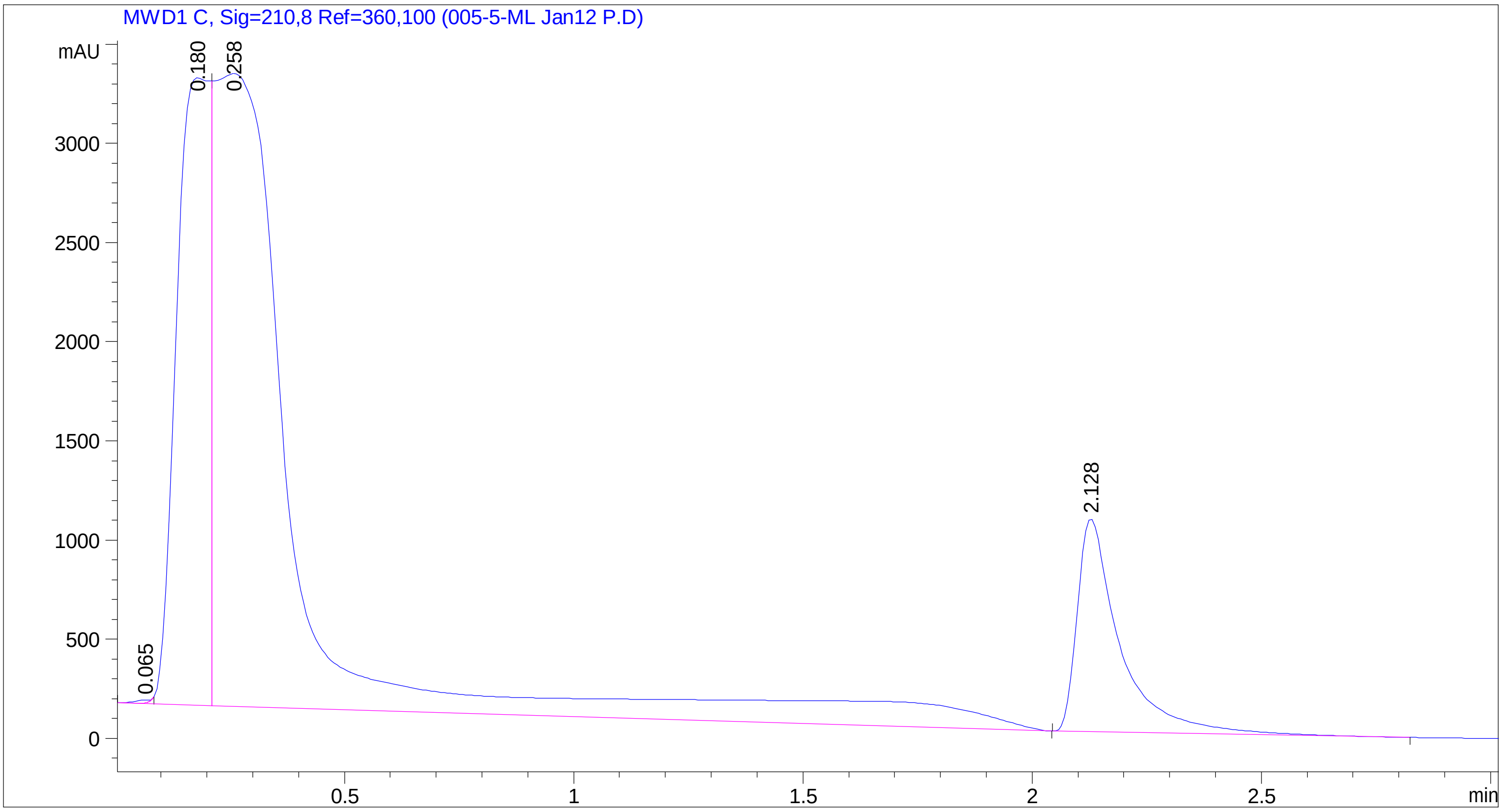

===

Area Percent Report

===

Sorted By : Signal
Multiplier : 1.0000
Dilution : 1.0000
Use Multiplier & Dilution Factor with ISTDs

Signal 1: MWD1 C, Sig=210,8 Ref=360,100

Peak #	RetTime [min]	Type	Width [min]	Area [mAU*s]	Height [mAU]	Area %
1	0.065	BV E	0.0357	34.75380	14.27425	0.0556
2	0.180	VV R	0.0767	1.59136e4	3163.62354	25.4499
3	0.258	VB	0.1747	3.95779e4	3190.16333	63.2951
4	2.128	BBA	0.0921	7002.86279	1073.53003	11.1994

Totals : 6.25291e4 7441.59115

===

*** End of Report ***
